# Insight into What Is inside Swift Heavy Ion Latent Tracks in PET Film

**DOI:** 10.3390/polym15204050

**Published:** 2023-10-11

**Authors:** Adil Z. Tuleushev, Fiona E. Harrison, Artem L. Kozlovskiy, Maxim V. Zdorovets

**Affiliations:** 1 Engineering Profile Laboratory, L.N. Gumilyov Eurasian National University, Astana 010008, Kazakhstan; adilzht@outlook.com (A.Z.T.);; 2 Laboratory of Solid State Physics, The Institute of Nuclear Physics, Almaty 050032, Kazakhstan

**Keywords:** PET film, swift heavy ion, molecular ordering, electrification of PET sample, hypsochromic shift of absorption edge

## Abstract

We present here a novel experimental study of changes after contact electrification in the optical transmission spectra of samples of both pristine and irradiated PET film treated with Kr^+15^ ions of energy of 1.75 MeV and a fluence of 3 × 10^10^ cm^2^. We used a non-standard electrification scheme for injecting electrons into the film by applying negative electrodes to both its surfaces and using the positively charged inner regions of the film itself as the positive electrode. Electrification led to a decrease in the intensity of the internal electric fields for both samples and a hypsochromic (blue) shift in their spectra. For the irradiated PET sample, electrification resulted in a Gaussian modulation of its optical properties in the photon energy range 2.3–3.6 eV. We associate this Gaussian modulation with the partial decay of non-covalent extended conjugated systems that were formed under the influence of the residual radial electric field of the SHI latent tracks. Our studies lead us to suggest the latent track in the PET film can be considered as a variband material in the radial direction. Consideration of our results along with other published experimental results leads us to conclude that these can all be consistently understood by taking into account both the swift and slow electrons produced by SHI irradiation, and that it appears that the core of a latent track is negatively charged, and the periphery is positively charged.

## 1. Introduction

The properties of latent tracks in polymer films irradiated with swift heavy ions (SHI) are of interest from a scientific and applied point of view and have long been studied by a number of researchers [1,2,3,4,5]. The formation of latent tracks in a thin film of polyethylene terephthalate (PET) irradiated with Bi ions of energy of 1.4 GeV using the so-called track-UV technique [6,7] is of particular note. Used as membranes, these films have extremely high indicators of both ion selectivity and permeability in aqueous salt solutions, and they act as ultrafast ionic sieves. These impressive experimental results have, however, highlighted shortcomings with the generally accepted understanding of the underlying physics, which lead to internal inconsistencies in explanations. For example, in [6,7], the authors conclude that the ultrafast ionic sieve is formed of sub-nanopores, with a diameter of less than 1 nm and negatively charged walls. The creation of these sub-nanopores is explained as being due to amorphization, destruction of molecular bonds, and a decrease in the density of the PET polymer in the central part of the track as a result of the outgassing of volatile products caused by the influence of swift electrons moving to the track periphery. Immersion in an aqueous electrolyte provides the negative charge on the surface of the subnanopores in the form of carboxylic COO− ions. Their electrostatic interaction with ions in aqueous solution results in the impressive selective properties of the ultrafast ionic sieve. According to the authors, the small transverse size of the subnanopores prevents the formation of a double electrical layer capable of shielding the electrostatic field of the COO− ions. It appears to be assumed that the rest of the PET film material is electroneutral. It is also clear that the authors interpret the interaction of SHI with the film using the model of *δ*-electrons and the secondary swift electrons caused by them [8,9], which for convenience we will call the “swift electron model”.

This explanation, however, contains internal inconsistencies. According to the swift electron model, electrons move out of the central part of the latent track, leaving it positively charged, taking the ion energy with them into their interactions with the molecular structure of the polymer in the track shell. Many studies of these interactions [9,10,11,12,13,14] focus solely on the energy transfer from the swift electrons that leads to radiation cross-linking of polymer chain molecules in the track shell, with no consideration of the negative electron charge transfer from the center of the track to its periphery.

In [15,16], the results of X-ray studies of U-irradiated thin polyimide and polycarbonate films show that the central zone has a radial size of about 3 nm: using the swift electron model, this is assumed to be electron depleted. The total volume charge created in a central zone of this size is significantly greater than the negative charge of a layer of carboxyl ions on the inner wall of a subnanopore.

Unless the swift electrons escape the film, the positive charge of the central zone of the latent track should be matched by a negative charge in the shell. Extensive industrial processing of PET films to produce membranes shows no evidence of net charge in films after SHI. There must therefore be a redistribution of electrons within the film and a consequent radial electric field.

Since PET is an electret [17,18,19,20,21,22,23], any internal field can persist for a very long time [15,16] and serve as an ordering factor for mobile groups within repeat units of PET chain molecules. Experimental confirmation of the internal redistribution of electrons is provided by our measurements of the electrostatic potential of PET films before and after irradiation with SHI fluences, reported below.

The interaction time of SHI with the polymer electronic subsystems has been shown to be no more than 10^−15^ s, and the lattice relaxation time of the resulting excitation to be up to 10^−11^ s [15,24,25]. Mobile groups in PET molecules with a dipole moment (terephthalate moieties) therefore have enough time to move in response to the presence of a radial electric field in the latent track in order to form a secondary ordered molecular structure. We reported experimental confirmation of the secondary ordering of the molecular structure of PET films after SHI irradiation in [26]. A later study using the method of thermally stimulated depolarization, which is often used to study electrets [27], provided confirmation of ordering by showing that the intensity of *α*-relaxation associated with orientation rotation of polar groups inside PET chain molecules in a film irradiated with Xe^+26^ ions and fluence of 4.5 × 10^8^ cm^−2^ is almost one order greater than that in the pristine state, and it disappears altogether after etching of the latent track to a transverse size of about 40 nm.

This ordering occurs in the amorphous part of the polymer film. As the density of polymers increases with ordering [28,29], this is inevitably associated with negative dilatancy: a decrease in the volume occupied by the set of molecules involved in the formation of the secondary ordered structure. The cylindrical symmetry of the latent track means that the increase in density of the ordered regions results in a decrease in the density along the central axis of the latent track until the appearance of a free volume.

We can estimate the dilatancy needed to form a free volume channel of the subnanopore size reported in [6,7] in a latent track of radial size of about 3 nm, as measured in [15,16]. We assume, as others do, that this central zone has a completely amorphous structure, as well as an initial density equal to the density of the monomer 1.33 g/cm^3^ [28]. If the partial ordering of the molecular structure due to the residual radial electric field caused an increase in density to 1.34 g/cm^3^, the volume reduction would be sufficient to form a free channel with a radius of 0.3 nm in the center of the latent track. This provides an alternative explanation for the appearance of a central subnanopore as reported in [6,7] that is consistent with the experimental results in [15,16].

We have consistently argued that a full explanation of SHI interactions with PET films enough must consider the electret properties of the polymer [26]. Our research led us to the conclusion that a PET polymer film irradiated with SHI provides a good basis for the industrial production of cheap unipolar electrets due to the simplicity of the spatial separation of charges in the cross-section of latent tracks. Bearing in mind the possible technological interest in this kind of product, on 17 May 2021, we claimed this as our intellectual property, now protected by the Kazakhstan patent no. 35709 “Method of forming of a film polymer electret”.

We have also consistently argued that the swift electron model is incomplete (hence why its application leads to internal inconsistencies). In the canonical explanation of Bethe and Ashkin [8], in addition to swift electrons, SHI irradiation also produces a larger fraction of slow electrons, with energies below the ionization potential of the medium. In contrast to the swift electrons, which are knocked out of the core of the latent track, the slow electrons are pulled into the core of the latent track by the electric field of the SHI. The net charge distribution in the central core is simply the sum of the knocked-out swift electrons and the incoming slow ones. According to [8], there are more slow electrons, so the residual charge in the core of the latent track will be negative, before interaction with electrolytes or solvents. The outer region of the latent track will acquire a volumetric positive charge. This is the opposite sign of charge redistribution to that predicted when only swift electrons are considered.

While the energy of the SHI is the key parameter for swift electrons, it is the charge of the SHI that is important for the slow ones. Experimental evidence [30,31] confirms that the post-irradiation state of substances irradiated by SHI depends on both the irradiating fluence and the ion charge. Whereas the effect of swift electrons on the molecular structure of the polymer involves breaking and re-formation of covalent bonds, the electric field created by the redistribution of electrons results in the formation of noncovalent dipole-dipole bonds between neighboring chain molecules. These are reversible, as has been confirmed experimentally [30,32].

We conclude that taking slow electrons into account as well as swift ones enables a consistent explanation of both the formation of an open channel and the presence of a negative volume charge in the core of the latent track, before immersion in an electrolyte. Investigation of the charge redistribution and the consequent internal electric field in PET films after SHI irradiation appears to us to be an interesting area for future research and experimentation. Studies of the shift of the absorption edge of PET polymer films after SHI irradiation [7,33,34,35] offer one promising approach. It is widely accepted that this red shift is caused by the growth of extended conjugated systems.

As explained above, the swift and slow electrons produced by SHI irradiation provide two growth mechanisms: the formation of covalent bonds between neighboring molecules (cross-linking) as a result of swift electron damage to molecular chains, and noncovalent dipole-dipole bonds formed by the ordering of terephthalate moieties under the influence of a residual radial electric field. The former are irreversible, while the latter can be weakened or destroyed by a reduction in the internal electric field strength, which should lead to a reduction in the red shift—that is, a shift towards shorter wavelengths—of the absorption edge of irradiated samples. The greater the change in the electric field, the bigger the shift will be, in line with [36,37], where the spectral dependence of optical absorption and the changes caused by variation in the electric field follow the same mechanism. Changes in the shape of the observed absorption edge can therefore provide information about changes in the distribution of charge and electric field in the polymer film. In this experimental study, we investigated the internal charge distribution in latent tracks in PET film after SHI irradiation.

## 2. Experiment

### 2.1. Irradiation and Sampling

An industrial roll of PET film Hostaphan Mitsubishi Polyester Film RNK12 (Mitsubishi Polyester Film GmbH, Wiesbaden, Germany) with a thickness of 12 µm was used for the study. Irradiation of the sample with Kr^+15^ ions of energy 1.75 MeV/u was performed at the DC-60 heavy ion accelerator in Astana, Republic of Kazakhstan, in normal geometry using the method described in [35]. According to [38], this energy exceeds that required for the ions to pass through the film. We used the results of our X-ray studies reported in [26,30] to determine the irradiation fluence needed to produce a high density of tracks but without significant overlapping. These results showed that there is an adequate linear correspondence between the magnitude of the SHI charge and the value of the latent track radii, enabling us to estimate the radius of the latent track of a Kr^+15^ ion as 26 nm, and hence the desired irradiation fluence as 3 × 10^10^ cm^−2^. The experimental fluence used was estimated using the technique and measurements described by us in ([35], see Figure 6). Samples for spectrometric studies were cut from the central part of the transverse cross-section PET roll before and after irradiation, keeping a record of the machine direction.

### 2.2. Initial Measurement of the Electrostatic Potential of the Film

The measurement of the electrostatic potential of the PET film before and after SHI irradiation was performed using an electrostatic capacitive probe IPEP-1 manufactured by OJSC MNIPI, Minsk, Belarus. The manufacturer’s specification states that the probe provides measurement of the potential of electrostatically charged objects at a distance of 10 cm from the measured surface in the range from 20 V to 2 kV with an error of about 50% at the lower limit of the measurement range. The distance during our measurements was 10 cm. The measurements were performed two months after irradiation of the PET film sample, and no residual static potentials were recorded in either pristine or irradiated samples. As a check that the device was functioning correctly, we noted that it recorded static potentials of a triboelectric nature that arose when moving samples. Based on these measurement results, we concluded that both the pristine and irradiated samples are electrically neutral.

### 2.3. Electrification of Samples

Since we are seeking to change only the charge state of the latent tracks in the irradiated film, we used a contact sample electrification scheme [17,23,39] at room temperature, placing the PET film between flat glass electrodes with a thick Au film coating that provides a tight fit to the surface of the film, thus minimizing the influence of the atmosphere and light on the samples during charging. We used these electrodes rather than evaporating metal electrodes directly to the surface of the films in order to avoid barrier effects at the film–metal interface that might cause changes to the charge state of the samples before electrification. We use the term electrification, following [39], because of the difference between our experimental scheme and that which is generally used in electret studies. See, for instance, [17,18,25,40]: in [40], the polarization of samples of PET films from the same manufacturer with a thickness of 12 to 50 µm was performed using aluminum electrodes and unipolar corona discharge at voltages up to 100 V for 5 min at temperatures above the glass transition temperature.

Since the peripheral regions of the latent track are positively charged and their projection onto the contact surfaces of the film is about two orders of magnitude greater than that of the core, we applied a negative dc voltage of 110 V to both electrodes for 48 h. This injects electrons into positively charged traps of various nature in the surface and deep layers of the irradiated PET film material. The main mechanism for electron transport into these regions is tunnelling of electrons from the electrodes through shallow localized states to places where positively charged holes are concentrated [17,39]. We performed the same experiment on a sample of the pristine PET film, where the distribution of positive charges by volume due to the presence of charge traps is expected to be generally uniform [17,18,22,23,25,39]. Five minutes after the electrification process was stopped, the residual surface potentials of both samples were measured. The average values for the irradiated sample were negative, around −45 V, and for the pristine sample, near zero (possibly a few volts with very low accuracy since this is well below the lower measurement limit of the probe used).

### 2.4. UV-VIS/near IR Spectroscopy

Measurements of the optical transmission spectra of samples of the pristine and irradiated PET films before and after electrification were performed using the Jena Specord-250BU analytical spectrophotometer (Analytik Jena, Jena, Germany) with the sample positioned so that the machine direction coincided with the direction of the spectrophotometer slit.

Cleaning of interference fringes from the observed spectral interferograms of the pristine and irradiated samples was performed using the technique given in [41,42] and described in detail in [35].

## 3. Results

A comparison of the transmission spectra of both pristine and irradiated samples cleaned of interference fringes showed that after contact with negative electrodes, there were shifts in the absorption edge towards the short-wave region that significantly exceeded the experimental measurement error. The presence of measurable changes in the transmission spectra after electrification confirms that electrons were injected into the film, due to the electric field between the electrodes and the internal positively charged regions of the film. Before discussing what these changes say about the distribution of electric fields within the samples, we show how the data collected in this experiment confirm and refine our previous results concerning the interaction of SHI beams with PET films.

**Figure 1:**
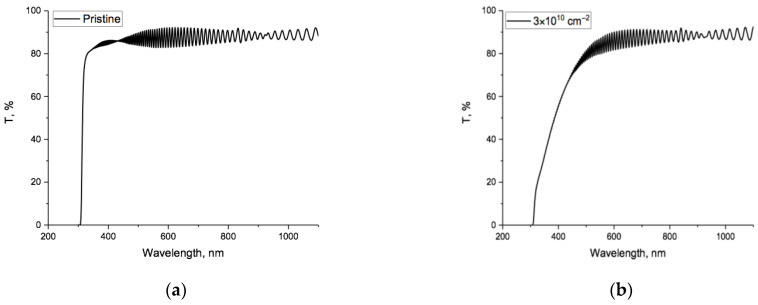

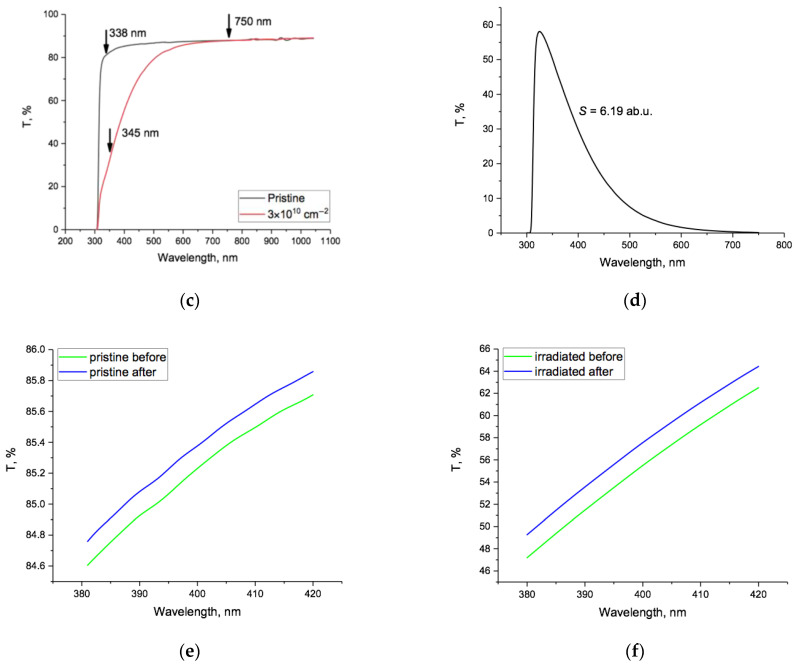
Experimental spectral dependences of the transmitted percentage of incident light, T%, of PET film samples: (**a**) pristine; (**b**) after irradiation; (**c**) spectra cleaned of interference fringes: before (black) and after irradiation (red); (**d**) the spectral difference function; (**e**) a section of the spectra of the pristine film before (green) and after electrification (blue); (**f**) the same for the spectra of the irradiated film. Note difference in vertical scale between (**e**,**f**), due to the difference in values of T%.

Figure 1a,b shows the observed UV-VIS/near IR transmission spectra of, respectively, the pristine and irradiated PET film samples. Figure 1c shows these two spectra cleaned of interference fringes across the spectral regions where fringes were present (marked with arrows).

It can be seen from Figure 1c that for wavelengths above 750 nm/1.65 eV (the red boundary of visible light) [43], the difference between pristine and irradiated spectra was less than 0.1%, and for this reason, we limited further consideration of changes in the spectra to the UV-VIS region only. Figure 1d shows the spectral difference function between pristine and irradiated samples across the UV-VIS region, where the red shift of the absorption edge occurred. In [35], we showed how the integral area under this curve could be used to estimate the irradiation fluence.

The transmission spectra for pristine and irradiated samples after electrification were likewise cleaned of interference fringes. Both showed a blue shift towards shorter wavelengths. Unsurprisingly, the changes were small and difficult to see against large transmission values across the full spectra, so Figure 1e,f shows sections of the spectra before and after electrification in the region 380–420 nm, for, respectively, pristine and irradiated samples. Note that the change due to electrification for irradiated film was an order of magnitude greater than for pristine spectra.

**Figure 2:**
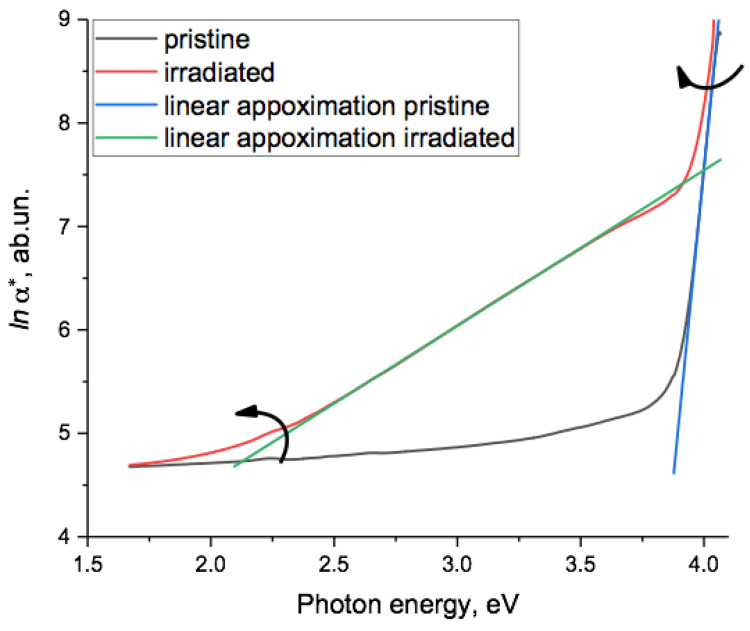
The function lna*(hν) for the spectra of the pristine (black line) and irradiated (red line) samples of PET film before electrification. The green line is the linear approximation lna*(hν) = 1.5031*hv* + 1.5315 with R^2^ = 0.9999 over the interval 2.5–3.5 eV for the irradiated sample. The blue line is the linear approximation for the pristine sample. The linear section in the spectrum of the irradiated sample at high photon energies is shown in Figure 3. The arrows show the direction of rotation of the two linear sections around upper and lower focal points when the SHI irradiation fluence was increased, as shown in [44] and explained in more detail in the text.

We have previously demonstrated that the logarithm of the non-transmittance function α*(hν) is a useful tool for the analysis of optical spectra of irradiated PET films [44]. Here, α*(hν) is the dependance of the sum of the intensities of both absorbed and reflected light on the photon energy *hν* and characterizes the extent of interaction between the film material and the incident light [45], including multiple reflections from the film–air boundaries [46]. Figure 2 shows lna*(hν) for the interference-free spectra of the pristine and irradiated film before electrification in Figure 1c.

**Figure 3:**
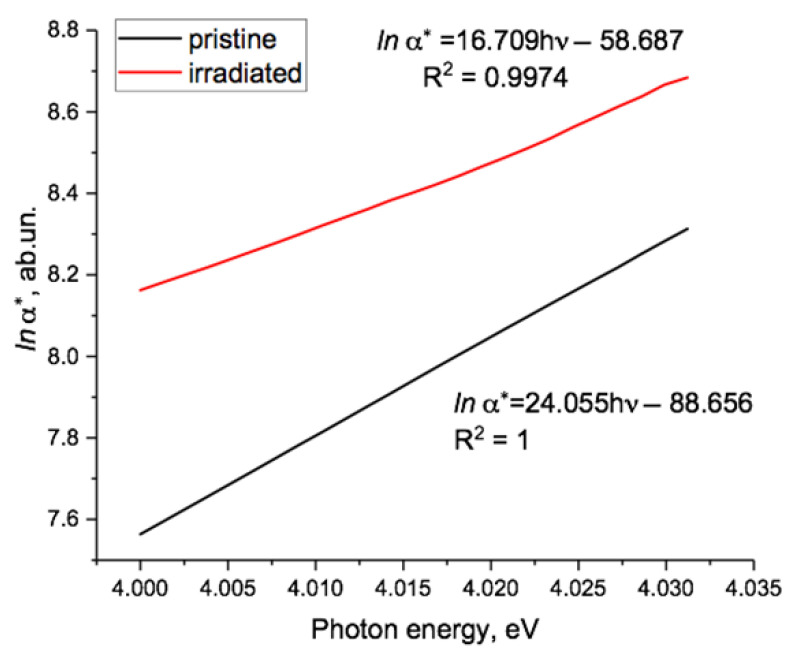
High energy section of Figure 2 showing the linear approximations of lna*(hν) for pristine and irradiated film, which intersected at the focal point at 4.08 eV.

The post-electrification graphs of lna*(hν) were very similar and are presented later. As can be seen, for the irradiated sample, there was a section in the region of 2.5–3.5 eV that was well approximated by a linear function and a shorter linear section in the spectra of both the irradiated and the pristine samples around 4 eV, shown in more detail in Figure 3.

In [44], we provided wider evidence for there being two linear sections in the function lna*(hν) for SHI-irradiated samples of PET film, reflecting two different exponential dependencies of α* on *hν* in two different photon energy regions. As the irradiation fluence increased, these two linear sections rotated in opposite directions, as shown by the arrows in Figure 2. The lower energy section rotated counterclockwise around a lower focal point at about 1.3–1.5 eV, and the higher energy section rotated clockwise around an upper focal point at about 4.1 eV. Comparing this result with established experimental and theoretical understanding of electronic processes in noncrystalline materials, we concluded that the focal point values were the bandgap values of the latent track material. As the bandgap at any particular point can only have one value, we attributed the upper value to the material of the electron-enriched core of the latent tracks and the lower one to the electron-deficient region of track peripheral zones.

Figure 3 shows in more detail the high photon energy region of Figure 2, where lna*(hν) was linear for both pristine and irradiated PET samples, confirming the Urbach nature of the absorption edge in the energy range around 4 eV. Linear extrapolation of the two experimental lines provided a focal point at 4.08 eV, characterizing the optical energy gap of the material, which correlated well with the results obtained in [19,21] and in our article [44]. Based on the results in [47], we interpreted the rotation of the Urbach line with increasing fluence found in [44] as a sign of increasing internal electric fields in PET films with increasing SHI irradiation.

**Figure 4:**
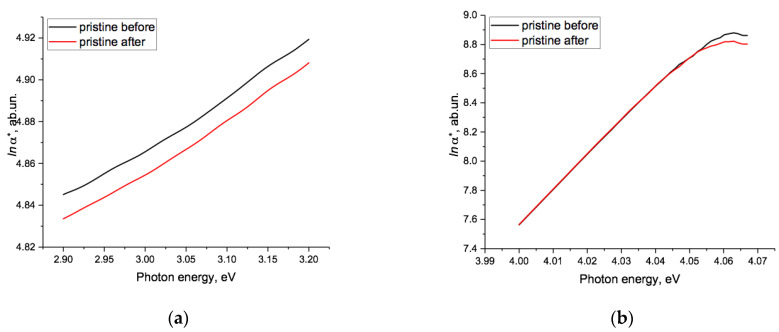
Changes in lna*(hν) for the pristine sample before (black line) and after (red line) the electrification process (**a**) over the energy range 2.9–3.2 eV and (**b**) for the range 4.02–4.07 eV.

The electrification process led to various changes in the transmission spectrum and hence to the function lna*(hν). Figure 4a,b shows details of the changes in the pristine film in two regions, the first (Figure 4a) over the photon energy range 2.9–3.2 eV, which corresponded to the region of high transmission, and the second (Figure 4b) around 4 eV, where transmission was dropping rapidly (see Figure 1). In the first region, electrification resulted in a generally uniform downwards shift of lna*(hν) to lower values of non-transmittance, but not of a linear Urbach type. In the second, lna*(hν) at this scale was almost unchanged by the electrification process and remained approximately linear up to a photon energy of about 4.04 eV, after which small oscillations were present both before and after electrification (though reduced). Above about 4.05 eV, lna*(hν) was reduced slightly by electrification. We discuss these changes later.

**Figure 5:**
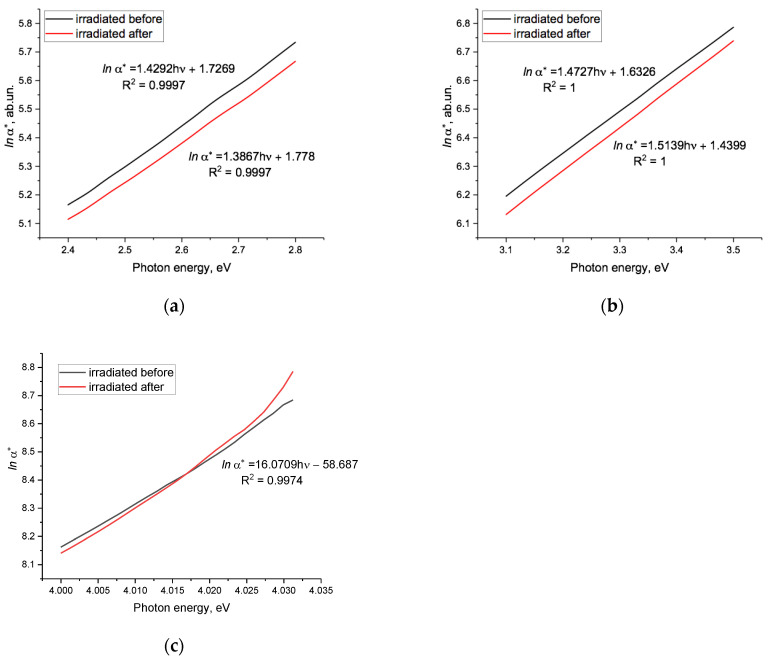
Function lna*(hν) for the irradiated sample before and after electrification: (**a**) in the range 2.4–2.8 eV; (**b**) in the range 3.1–3.5 eV; (**c**) in the range 4.00–4.03 eV. The black line is before electrification, red line is after electrification. The focal points for the straight lines in Figure 4a,b were at energies of 1.2 and 4.68 eV, respectively. Note the different vertical scales.

Figure 5 shows lna*(hν) before and after electrification for the irradiated PET sample. Due to the small but varying size of the changes, Figure 5a–c shows the enlarged sections of lna*(hν) in the regions of 2.4–2.8 eV, 3.1–3.5 eV, and 4.00–4.03 eV (note the different vertical scales). These show that after electrification, in the two lower energy regions, lna*(hν) remained linear and of the Urbach type, though of different inclination in the two regions. The general shifts that occurred were not dissimilar to those in the pristine sample, but the downwards shift of lna*(hν) was approximately half an order magnitude larger than that in pristine film (see Figure 4a). The emergence post-electrification of two distinct linear parts across the range from 2.4 to 3.5 eV, with different focal points and directions of rotation (as can be seen from the *hv* coefficients), is discussed further below.

The position of the lower focal point at 1.2 eV is in reasonable accordance with the result in [44] (where it was found to be 1.3 eV–1.5 eV). The position of the upper focal point at 4.68 eV is somewhat higher than the value of 4.1 eV found in Figure 3 and earlier. The directions of rotation around the upper and lower focal points after electrification are opposite to those resulting from an increase in the SHI fluence [44], as well as Figure 2 above. Since the experimental electrification scheme used in our experiment led to a decrease in internal electric fields, the direction of rotation of the linear approximations to sections of lna*(hν) around the focal points served as an experimental signature of the increase/decrease in the average amplitude of internal electric fields in the PET film as a result of exposure of one kind or another. The latter is in agreement with the conclusions in [47,48,49].

We will leave the study of this discrepancy for the future, but note that since the non-transmittance function of lna*(hν) was the total value of the absorbance and reflection of light when passing through the sample under study, the discrepancy may have been due to the influence of a reflection peak noticeable near this energy in the reflection spectrum of a thick (47 µm) PET film [19], which is also represented in the absorption spectrum of benzene [50]. At this energy, well-pronounced peaks of absorption and photo-induced conduction current were also found in a very thin (<1 µm) PET film [21], which may indicate the participation of the benzene ring in the accumulation of extra electrons during our contact electrification of the PET film [18].

Despite this discrepancy, these results show that the direction of rotation of the Urbach lines corresponded to growth/reduction of internal electric fields, and this also confirmed (in a different experimental scheme to [44]) the existence of two different focal points and, consequently, two different values of the bandgap, in the cores and shells of latent tracks in SHI irradiated PET films.

It can be seen in Figure 5c (red line) that, after electrification of the irradiated sample, lna*(hν) became over-exponential for photon energies above 4.02 eV, in the region of high non-transmittance, suggesting the presence of a resonant effect. This behavior was not present in the spectra of the pristine film (Figure 4b, red and black lines) or in the spectrum of the irradiated film before electrification (Figure 5c, black line), all of which showed good Urbach-type behavior around this energy. We therefore attributed it to the injection of electrons from the electrodes into the irradiated film.

Accumulation of additional *π*-electrons by the benzene rings in the terephthalate moieties of the PET film during the electrification process may provide the basis for a resonance mechanism. We have previously shown [26,30,32] that the red shift in the absorption edge of PET films after SHI irradiation is due to extended conjugated systems formed by the dipole–dipole interaction of terephthalate moieties of repeat units of neighboring PET chain molecules. The π-electrons in these systems are delocalized along the molecular conformations connected by the dipole–dipole interaction along the axis of the latent tracks, and in this respect have some similarities to metal-like nanoparticles. If some of the electrons injected into the PET film during electrification are absorbed by benzene rings in extended conjugated systems, we might expect to see an increase in metal-like behavior. In [51], it is shown that metal nanoparticles can interact with electromagnetic waves of much larger wavelength producing resonance effects. In our experiment, any such effect would only be detectable at low values of light transmission. The over-exponential behavior seen in Figure 5c might therefore be due to resonant light scattering by extended conjugated systems with higher densities of delocalized electrons.

Another possible explanation for this over-exponential growth of non-transmittance is the behavior of the electron–hole pair created by exciton ionization. As explained in [52], despite the absence of a Coulomb barrier, immediately after ionization, the electron is held by classical forces in a resonance-like state in the hole’s potential, which can be responsible for a strong resonance in the optical absorption spectrum. Studies of the optical spectra of SHI-irradiated PET films thinner than those used in our experiment in the region of high non-transmittance after additional injection of electrons may provide further insight into this behavior.

**Figure 6:**
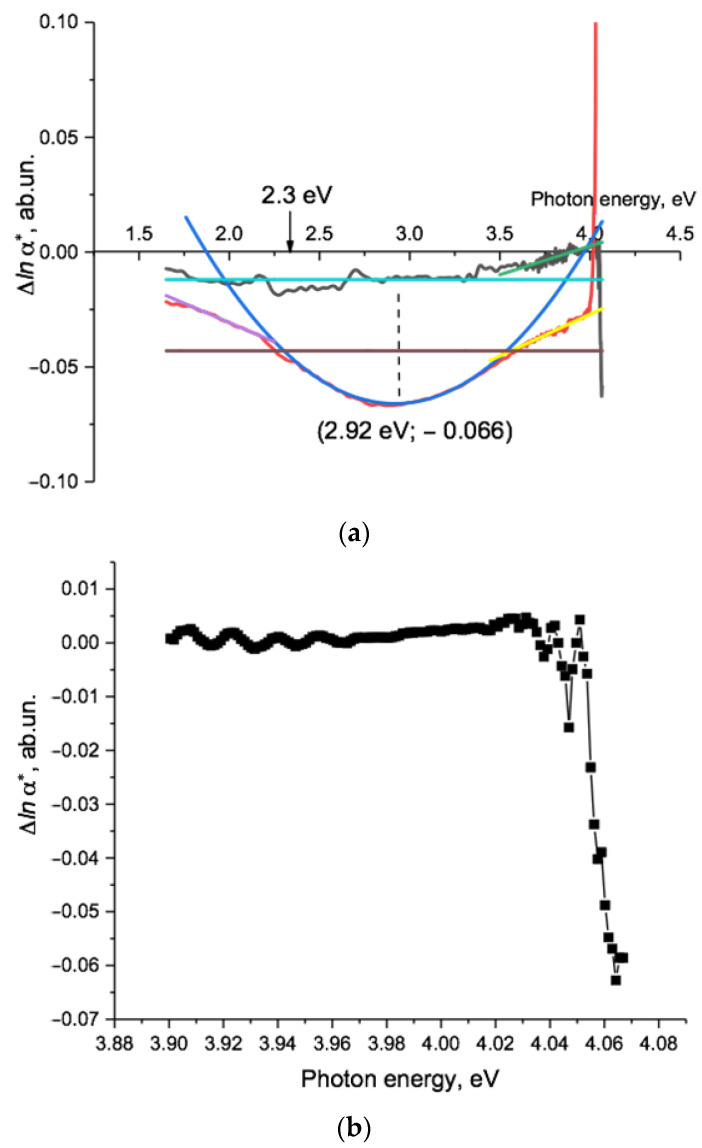
(**a**) Difference function Δlna*(hν) of PET film samples before and after electrification. The black line is the pristine sample; the red line is the irradiated sample. The light blue line shows the average value of Δlna*(hν) = −0.012 for the pristine sample over the energy range of 1.65–3.5 eV. The brown line shows a similar value of Δlna*(hν) = − 0.043 for the irradiated sample at an energy range of 1.65–3.95 eV. The dark blue line is a quadratic approximation of Δlna*(hν) = 0.0603*(hν)*^2^ − 0.3522*hν +* 0.4481 = 0.0603(*hν* − 2.92)^2^ − 0.066 with *R*^2^ *=* 0.9916, obtained from the experimental points of the Δlna*(hν) spectrum of the irradiated sample in the energy range 2.35–3.5 eV. The magenta and yellow lines indicate linear approximations Δlna*(hν) = 0.0336 *hν* + 0.0365 with *R*^2^ *=* 0.994 in the interval of 1.77–2.1 eV below and Δlna*(hν) = 0.0363*hν* − 0.1723 with *R*^2^ *=* 0.947 in the interval of 3.6–3.8 eV above the area of quadratic dependence. The green line indicates the linear approximation Δlna*(hν) = 0.0249*hν* − 0.097 with *R*^2^ *=* 0.7 in the interval of 3.4–4.0 eV. The low value of *R^2^* was due to the presence of oscillations on the experimental line, the nature of which needs an additional study. (**b**) Expansion of the difference function Δlna*(hν) of the pristine sample shown in Figure 6a for the high energy region of 3.9–4.07 eV. At this expanded scale, it is possible to see the individual experimental data points (squares), which appear as a smooth curve in Figure 6a.

We now use a difference method to analyze in more detail the effect of electrification on the non-transmission function [46]. Figure 6a,b shows the difference function Δlna*(hν), which is the logarithm of the ratio of α*(hν) values before and after the electrification process, obtained by subtracting the functions lna*(hν) of samples before electrification from those after. For the pristine film sample, the difference function Δlna*(hν) (black line, Figure 6a) was negative and broadly independent of photon energy up to about 3.4 eV (365 nm). Above this energy, the dependence on photon energy became approximately linear (the green line in Figure 6a) up to around 4 eV, the region where the value of Δlna*(hν) reached zero. The light blue horizontal line showed the position of the arithmetic mean value of Δlna*(hν), which was −0.012 in the interval of 1.65–3.4 eV. This corresponded to a ratio of α*(hν) values of 0.988, so the non-transmittance of the film decreased by 1.2%. The value of Δlna*(hν) lay below the mean across the region of photon energies between 2.3 and 2.6 eV, where phosphorescence was observed in [53]. The energy of the lower boundary of this region corresponded to the energy of a deep charge trap found in [54].

The negative value of the difference function and the nature of its dependence on photon energy reflect the fact that, as can be seen in Figure 1e and Figure 4a, electrification results in a parallel shift of the transmission spectrum towards shorter wavelengths/higher photon energies and a sharpening of the absorption edge. The only causal mechanism we can identify is the injection of electrons into the PET film sample volume due to an electric field between the negative electrodes and randomly distributed positive volume charges inside the film, resulting in the decrease in the internal random electric fields [47,55].

This explanation implies that the magnitude of the parallel shift seen in our experiments is a measure of the change in the root-mean-square value of the microscopic field. Larger values of the mean value of Δlna*(hν) are the experimental signature of larger post-electrification changes of local electric fields and, consequently, larger pre-electrification fields.

The broadly linear nature of Δlna*(hν) over the range 3.4–4 eV in response to a reduction in the internal electric field demonstrated that the changes in the spectral dependence of lna*(hν) after electrification, which are small and not easy to characterize, resulted from a counterclockwise rotation around a focal point at about 4 eV. The photon energy range 3.4–4 eV was where the (highly nonlinear) absorption edge lay in the transmission spectrum (Figure 2). Our explanation that the sharpening of the absorption edge is due to a reduction in the electric field in the post-electrification PET film is consistent with Dow and Redfield, who in [52] showed that broadening/sharpening of the absorption edge is a result of the dependence of an exciton absorption band on increasing/decreasing electric fields. The lower boundary of this band is difficult to identify from the transmission spectra, but it can be seen in the difference function: in pristine PET, the exciton absorption band lies between 3.4 eV and 4 eV. The absorption strength decreases slightly after electrification, and (in line with Dow and Redfield) we observed this decrease as a rotation of the spectral function lna*(hν).

In the upper part (above 3.88 eV) of this broadly linear region of Δlna*(hν), there were a series of oscillations, shown in close up in Figure 6b.

There were four low-intensity waves with amplitudes from 0.001 to 0.003 (0.1–0.3%) with minima at 3.9154, 3.9315, 3.9465, and 3.9629 eV, where Δlna*(hν) was slightly below zero. The gaps between these weak additional absorption bands were approximately 16 meV.

At even higher photon energies, there were three further minima at 4.0378, 4.0470, and 4.0642 eV. The last of these had a very large negative value of Δlnα*(hν) = −0.063. This was more than five times its average value of −0.012 in the lower energy region (light blue line in Figure 6a) and corresponded to a 6.1% change in the amplitude of the non-transmittance after electrification.

Sharp peaks of light absorption by amorphous dielectrics and semiconductors below the absorption edge in [48,55,56,57,58,59,60] were associated with exciton absorption lines whose maxima corresponded to the minima of the difference function obtained by subtracting the initial spectra from the spectra after electric field modulation [61]. This suggests that the observed minima in Δlna*(hν) (see Figure 6b) were associated with exciton absorption bands. Measurements on films that are at least half as thin are needed to study the fine structure of non-transmittance in the area of the absorption edge in more detail.

For the irradiated PET sample, the difference function Δlna*(hν) (Figure 6a, red line) was generally more negative than for the pristine sample. The brown horizontal line shows the arithmetic mean of Δlna*(hν) = −0.043, which corresponded to a decrease in non-transmittance of 4.2%. This was more than three times the change for the pristine film, reflecting the fact that before electrification, the SHI irradiated PET film sample had a higher internal electric field than the pristine sample.

Across the energy range 1.65–3.95 eV, the spectral dependence of Δlna*(hν) can be divided into three parts. In the lower and upper regions of 1.75–2.15 eV (708–577 nm) (red wing) and 3.6–3.95 eV (344–318 nm) (blue wing), Δlna*(hν) was broadly linear (magenta and yellow lines and equations in caption to Figure 6a show linear approximations). Although of similar form (but opposite gradient), the two wings differed quantitatively: the value of Δlna*(hν) on the lower border of the red wing was −0.02 (2%), whereas that on the upper border of the blue wing was −0.029 (3%). A larger change indicated a larger initial field before electrification, and as we have previously linked the spectrum in the higher photon energy region to the inner core of latent tracks (see above, Figure 2), this indicates that the initial field in the core was larger than in the periphery.

As with the pristine film, the use of the semilogarithmic difference function Δlna*(hν) simplified changes to complex transmission/non-transmittance spectra and enabled the changes to be understood as rotations around focal points. Here, these were, respectively, at 4.75 eV and 1.09 eV for the blue and red wings, and (as with the pristine sample) the directions of rotation were opposite to those seen with increasing SHI irradiation fluences. The energy of the upper focal point here coincided with the 260 nm benzene absorption line mentioned in the discussion of the graphs in Figure 5, providing further confirmation that benzene rings in the extended conjugated systems created by SHI irradiation are involved in the absorption and storage of extra electrons.

The energy of the focal point of the red wing was near the range of lower focal points that we have previously observed [44] as having values that depend on the specific irradiation conditions. In the region between these two wings, their linear dependencies evolved smoothly into a quadratic dependence of Δlna*(hν) with a minimum at the point (2.92 eV; −0.066), where the decrease in non-transmittance was 6.4%. The equation for the best fit quadratic (dark blue line) is given in the caption to Figure 6a.

This quadratic dependence of the semilogarithmic difference function Δlna*(hν) corresponded to a modulation of the pre-electrification non-transmittance function α*(hν) by *exp(*−*ν^2^)* — that is, a Gaussian function. This region corresponded to the region of good linear Urbach dependence of lna*(hν) for the irradiated sample before electrification (Figure 2, green line). It is well known that Gaussian (or normal) distributions characterize stochastic variables that are the sum of the values of a large number of small and weakly interdependent physical processes [62]. We conclude that the observed negative value and quadratic dependence of Δlna*(hν) was a manifestation of a reduction in the probability density of optical transitions in noncovalent extended conjugated systems in the irradiated PET sample after its electrification. Using the quadratic equation given in the caption in Figure 6a, we can calculate the standard deviation *σ* of the Gaussian as 2.88 (≈3), which indicates that it (and therefore the change in non-transmittance after electrification) arises from *one* physical property of the latent track material. This is the change in the ordering of the terephthalate moieties of the PET chain molecules in response to the reduction in the internal electric field caused by the electrification process.

Finally, in the region of the highest photon energies above 4 eV, Δlna*(hν) increased sharply, becoming positive above 4.01 eV (when the post-electrification non-transmittance of the irradiated sample exceeded the pre-electrification value, as can be seen in Figure 5c). As discussed above, this suggests the emergence of some resonant process.

## 4. Discussion

1. Our electrification scheme was designed to avoid contact/barrier effects such as those encountered when evaporating metal electrodes onto surfaces. It is well known that a PET film/Au electrode pair is close to neutral in the triboelectric sense: that is, when a PET film comes into contact with an Au electrode, no contact electrification associated with the difference in the work function occurs [63]. Studies of photocurrent spectra in thin PET films have also shown that Au electrodes make a negligible contribution to electron photoinjection [21]. We therefore conclude that the observed changes in the optical spectra of our PET film samples after electrification are due to electrons moving into the PET film from the electrodes under the influence an electric field between the (negative) electrodes and (positive) volume charges inside the film.
2. The exponential absorption edge in semiconductors and insulators in the high energy region immediately below the bandgap (known as the Urbach edge) has been extensively studied and its electro-optical nature is well established. In semi-logarithmic coordinates, gradients of between 5 and 22 eV^−1^ for the Urbach edge in this region are characteristic of many inorganic amorphous semiconductors and dielectrics [48,52,55,56,57,58,59]. For example, the amorphous semiconductor IV-compound Ge_16_As_35_Te_28_ Si_21_ has an Urbach edge gradient of 22 eV^−1^ and Se one of 17 eV^−1^. The values of 24 eV^−1^ and 17 eV^−1^ found here for the Urbach line in the high energy region in pristine and irradiated PET films, respectively (see Figure 3), were consistent with these other results. In a number of amorphous semiconductor glasses, there is another noticeably flatter exponential region of the spectrum at lower energies further below the absorption edge, with semi-logarithmic gradient values of 0.64–3.3 eV that depend on the structure and history of the material [48]. The gradient of this lower energy Urbach line for our irradiated PET sample was 1.5 eV^−1^ (see Figure 2, green line), which lay in the middle of this interval. Together with the established semi-crystalline nature of the PET film, these results suggest that the theory of electronic processes in noncrystalline materials is applicable here [56].

According to Mott’s theory of band-state densities in amorphous materials, the absorption of photons with energies less than the optical bandgap is associated with the presence of tail states in the gap, and both the upper and lower exponential sections of the broadened absorption edge are determined by the transitions between localized states and extended states. In such materials, the bandgap experiences perturbations due to two types of fluctuations that are long range relative to characteristic atomic/molecular distances. One type is associated with fluctuations in the fields of charged centers (electrostatic part), and the other with fluctuations in the density of the material (deformation part). Both have exponential distributions of localized states in the gap. Mott’s theory predicts that electrostatic fluctuations have a weak effect on the width of the optical bandgap, whereas density fluctuations strongly affect it. In practice, observed fluctuations generally comprise both types of fluctuation in different proportions in different parts of the material [48,49,52,56,57,58]. The use of semi-logarithmic coordinates, in which exponentials are represented as straight lines, enables the contributions of the two types of fluctuation to be distinguished. It is then possible to see the separate contributions to the broadening of the absorption edge arising from changes in the charge state, as well as changes in the molecular structure, in PET films after SHI irradiation.

3. Using Mott’s theory of band-state densities in amorphous materials, the uniform decrease in the non-transmittance of semi-amorphous pristine PET film after electrification for energies up to 3.4 eV (as seen in Figure 6a) can be explained as due to a decrease in the density of holes in the tail states in the bandgap near the top of the valence band, which leads to a decrease in light absorption. In other words, the electrification process has led to the neutralization of a number of positively charged centers in the surface layers and within the PET film, which can be estimated by the change in light absorption. Due to the generally uniform distribution of charged centers throughout the pristine PET film, the electrification process leads only to a change in the RMS of the random field in the amorphous part of the PET film, manifested as electrostatic bandgap fluctuations. We intend to carry out further studies of the effects of varying the voltage and duration of electrification on the distribution of charged centers and regions in the PET film, as we believe these will help in understanding its electret nature.

Although semi-amorphous pristine PET film shares some similarities with amorphous semiconductor materials, there are also significant differences. These include the isolation of dipole terephthalate moieties from the rest of the PET chain molecule, the weak van der Waals interaction between neighboring chain molecules, and the presence of charge traps of various natures. These properties, together with the random orientations of dipole groups in the amorphous part of the PET film, allow us to consider the individual dipole terephthalate moieties as interacting with a homogeneous polarized medium, making it possible to compare the electrification process with the interaction of molecules with solvents [64]. In the latter, changes in polarization are provided by changing the solvent, whereas we change the polarization of the environment by injection of electrons into the PET polymer matrix.

In [64], a general empirical rule is established from a large body of experimental results from studies of the optical properties of dipole organic molecules in solvents. For such molecules, an increase in the dipole force leads to a bathochromic (red) shift, and a decrease leads to a hypsochromic (blue) shift. The hypsochromic shift that we observed after electrification (Figure 1) was likewise due to a decrease in the dipole force due to electron injection into the PET film. The dependence of shifts in the absorption edge on the strength of dipole forces is consistent with the conclusion in [21] that the position of the absorption edge at 4.1 eV in pristine PET film is already the result of the spreading of the benzene ring absorption line at 5.5 eV, as a result of resonant interactions with adjacent dipole carbonyl groups. We note here that, according to [65], in this context “resonance” is synonymous with “mesomeric effect” and is associated with the redistribution of delocalized electrons in a conjugated *π*-orbital system, rather than the more usual concept of resonance as the matching of oscillation frequencies of systems or parts of systems with some external driving force.

4. The difference function Δlna*(hν) for the irradiated PET sample (Figure 6a) reflects the changes in the state of the latent track material that occurred as a result of electrification, but not the prior changes in the PET film due to SHI irradiation, which we discussed in [44]. In the introduction, we showed that in both the “swift electron” model, and the “swift + slow electron” model, there was a redistribution of electrons between the core and shell of the latent track, with the consequent appearance of a radial electric field, which was strongest in the transitional region between the core and shell. The two models predicted opposite field directions, but for both, the degree of ordering of terephthalate moieties was greatest in this transitional region. It follows that the biggest electrification-induced decreases in the internal radial field and in the degree of ordering of terephthalate moieties will be here as well. Disordering in its turn involves the scission of parts of spiral conformations, which had been in equilibrium with the latent track radial electric field before electrification, causing fluctuations in the density of the material and affecting the size of the bandgap in the transitional region. As described above, this disordering was reflected in the Gaussian modulation *exp(*−*ν^2^)* of the pre-electrification non-transmittance function α*(hν) in the region 2.3–3.6 eV, which was where we saw the biggest change in non-transmission after electrification, and thus the biggest change in the size of the bandgap.

We previously identified two different bandgap values for the material of the latent tracks in the core and periphery as given, respectively, by the upper and lower focal points around which Urbach lines rotated [44]. Now we can see how these could be connected by the behavior of the non-transmittance function in the energy region between these two bandgap values, if we assume that the Gaussian spectral region is associated with the transitional region between core and periphery. This would imply that the PET material within a SHI latent track is a type of variband material, such as those attracting the attention of researchers with their unusual properties (see for example [66,67]). This could be confirmed by additional experiments using an electrification scheme similar to the one in this study, but with variable electrode potentials and electrification times.

On each side of the quadratic dependence of the central part of the difference function Δlna*(hν) for the irradiated PET sample, there were two linear sections, in the photon energy ranges 1.77–2.1 eV (“red wing”) and 3.6–38 eV (“blue wing”) (Figure 6a: magenta and yellow lines, respectively). These reflect the exponential nature of the change in the probability density of optical transitions after electrification. Since we associated the quadratic region with one of the two types of fluctuation that lead to bandgap perturbation in Mott’s model (deformation type), we associate linear regions with the other, electrostatic, type of fluctuation. The pristine film showed similar linear behaviors in the energy range above 3.4 eV (Figure 6a, green line), which we likewise associate with electrostatic fluctuations.

The comparable values of the gradients of the linear sections of Δlna*(hν) for irradiated and pristine PET samples at high photon energies (Figure 6a, yellow and green lines, respectively), the stable position of the upper focal point when pristine film is irradiated with various SHI fluences and after electrification, and the observation of optical benzene properties in Δlna*(hν) for the irradiated PET sample indicated that for both pristine and irradiated samples, the responses in this high energy part of the spectrum were due mainly to the field of the terephthalate moieties. The higher gradient of Δlnα*(hν) for the irradiated sample was due to the SHI latent track field, in accordance with [47,55].

In the low energy part of the spectrum, the experimental results show that the position of the lower focal point varied according to the level of SHI irradiation and electrification. This dependence on external factors was in contrast to the upper focal point, whose stability indicated that it was due to intrinsic properties of the material. This dependence could be useful for future applications since it offers the opportunity to create material of variable optical bandgaps in the periphery of latent tracks.

These results highlight the significantly different physical nature of covalent and noncovalent changes in the molecular structure of PET films induced by the passage of swift heavy ions. According to [68], cross-linking takes place due to the influence of swift electrons and, in the main, chemically active light radiolysis products (free radicals) of chain molecules generated in the immediate vicinity of the ion trajectory and diffused to the periphery of the latent track. Chain molecules that have undergone cross-linking form extended conjugated systems based on covalent bonding. This process is purely stochastic in nature. In [68], the authors note that cross-linking of molecules leads to a decrease in molecular mobility, one of the consequences of which is the well-known fact that the etching rate of the shell material of the latent track is lower than that of the pristine film.

Extended conjugated systems formed by covalent bonds cannot be broken by the electrification scheme we used here. The scission we found associated with the blue shift after electrification of the PET absorption edge, previously red-shifted by SHI irradiation, must be due to the breaking of weaker noncovalent bonds, arising from dipole–dipole interactions of neighboring repeat units of PET chain molecules to form spiral conformations. It is well established that the SHI-induced red shift of the absorption edge was due to the growth of extended conjugated systems: our results show that at least some of these were formed by noncovalent bonds. The nature of the ordering/disordering of terephthalate moieties in the chain molecules of the amorphous part of the PET film and the subsequent formation/decay of spiral conformations was determined by the high mobility of these segments in the radial electric field of the latent track due to their strong dipole moment and σ-hinges. All terephthalate moieties will either be involved in noncovalent dipole-dipole interactions, or in covalent cross-linking. The ratio between these is a topic for further investigation.

Finally, as we were finishing this article, we became aware of an interesting experimental result presented in a recently published article [69], which, in our view, can serve as confirmation of the results of this study. In this article, the authors used a Hitachi SU8020 field emission scanning microscope (FESEM) to obtain micrographs of fractured PET film samples after irradiation with SHI and subsequent long-term treatment (up to 1000 h) with light from a low-pressure mercury fluorescent lamp with a suppressed short-wave part of the spectrum below 315 nm. Before using the FESEM, the PET film surfaces were decorated using the sputtering technique with a 10 nm thick Au-Pd layer. In a series of micrographs, parallel homogenous grey stripes in a matrix of alternating bright and dark grains of similar sizes were found.

The grey stripes were interpreted by the authors as areas of amorphization of the latent track material and the bright and dark grains in the surrounding areas as, respectively, crystallite and amorphous regions in parts of the PET film that were not exposed to the SHI. Using an image analysis program on a selected fragment, the authors determined that the peripheral parts of analyzed stripes were noticeably lighter, and the central part darker, than the average brightness value of the micrograph fragment. The grey stripes covered an area that was much larger than the amorphization zone according to previous data. The authors hypothesize that the relaxation process of internal stresses on the fault surface leads to a widening of the amorphization zone.

In the absence of any technical details of the micrograph process in [69], there are, however, other possible interpretations of the observed patterns. The well-known electret properties of PET film lead to the presence of microregions of different polarities [17,39] and consequently to the formation of an electric microrelief on its surface. This relief is a picture of electric fields between microregions of the substrate with different electrical properties and is the factor that has a decisive influence on the morphology of the growing film, not the geometry of the surface [70,71,72]. SEM studies of thin metal films growing on dielectric substrates with voltage reliefs have shown that the degree of brightness of the SEM micrographs reflects the charge sign of the microregion of the substrate, due to the different growth rate of the film on areas with different potential signs [72]. According to this book, areas where the substrate has a positive charge look bright and those with a negative charge look dark. The manufacturer of the FESEM Hitachi SU 8020 microscope used in [69] to examine the fractured SHI-irradiated and light-treated samples of PET film states that it has a routine capability for voltage contrast imaging. This allows the visualization of differences in the surface potentials of microregions of a sample under study, revealing its electrical microrelief [73].

In our opinion, this provides an alternative interpretation of the FESEM micrographs in [69] as showing the distribution of electric fields on the fractured PET surfaces. The bright and dark grains are due to electric domains with, respectively, positive and negative charges in areas of the PET film unaffected by the SHI irradiation. The homogenous grey of the latent tracks shows that the Coulomb field of the SHI has affected the electric domain structure, resulting in the latent track being a single charge system with cylindrical symmetry. In accordance with [72], since the peripheral regions of the latent track are brighter, they have a positive charge, and since the core is darker it has a negative charge. This corresponds with our assessment [35,44] that the peripheral parts of SHI latent tracks in PET film are electron depleted, and the inner core electron enriched.

We also note that the authors of [69] estimated the width of the latent track from the brightness profile by using the spectral measure of full width at half maximum (FWHM). The brightness profile, however, has a spatial rather than spectral character, so the FWHM of the dark minima below the average in the brightness profile is less than half of the width of latent track (as can be seen from the micrograph itself), and indeed the quoted value of 28 ± 4 nm for Kr ions is close to the value of 22 nm found by X-ray methods for the radius of the latent track in PET film irradiated with Kr^+14^ ion with an energy of 100 MeV [30].

## 5. Conclusions

Using the internal charged regions of the PET film samples as the second electrode for electrification provided further insight into the nature of the ordering of the molecular structure in latent tracks after SHI irradiation. The injection of electrons into the film by an electric field between the negative electrodes on the surface and the positively charged inner regions reduced the internal fields in the film. This led to the partial decay of expanded conjugated systems, previously formed by the residual electric field of the SHI latent tracks, manifested in the hypsochromic (blue) shift of the absorption edge after electrification. In semi-logarithmic coordinates, the value of the non-transmittance function α*(hν) of the irradiated PET sample after the electrification process was found to be a modulated Gaussian function of the photon energy in the range 2.3–3.6 eV.

Applying Mott’s theory of non-crystalline solids to our experimental results led us to suggest that in the radial direction, the latent tracks in the PET film are a variband material.

Analysis of experimental results from published sources led us to conclude that these can be consistently understood by considering both swift and slow electrons, and this indicates that the core of a latent track is negatively charged and the periphery is positively charged.

## Data Availability

The data presented in this study are available on request from the corresponding author.

## References

[1] Husaini S.N., Zaidi J., Malik F., Arif M. (2008). Application of nuclear track membrane for the reduction of pollutants in the industrial effluent. Radiat. Meas..

[2] Fink D. (2013). Fundamentals of Ion-Irradiated Polymers.

[3] Liu F., Wang M., Wang X., Wang P., Shen W., Ding S., Wang Y. (2018). Fabrication and application of nanoporous polymer ion-track membranes. Nanotechnology.

[4] Wang M., Shen W., Ding S., Wang X., Wang Z., Wang Y., Liu F. (2018). A coupled effect of dehydration and electrostatic interactions on selective ion transport through charged nanochannels. Nanoscale.

[5] Apel P.Y. (2019). Fabrication of functional micro- and nanoporous materials from polymers modified by swift heavy ions. Radiat. Phys. Chem..

[6] Wen Q., Yan D., Liu F., Wang M., Ling Y., Wang P., Kluth P., Schauries D., Trautmann C., Apel P. (2016). Highly Selective Ionic Transport through Subnanometer Pores in Polymer Films. Adv. Funct. Mater..

[7] Wang P., Wang M., Liu F., Ding S., Wang X., Du G., Liu J., Apel P., Kluth P., Trautmann C. (2018). Ultrafast ion sieving using nanoporous polymeric membranes. Nat. Commun..

[8] Bethe G.A., Ashkin Y., Segre E. (1953). The Passage of Radiation through Matter. Experimental Nuclear Physics.

[9] Bouffard S., Gervais B., Leroy C. (1995). Basic phenomena induced by swift heavy ions in polymers. Nucl. Instrum. Methods Phys. Res. Sect. B Beam Interact. Mater. Atoms.

[10] Apel P., Schulz A., Spohr R., Trautmann C., Vutsadakis V. (1998). Track size and track structure in polymer irradiated by heavy ions. Nucl. Instrum. Methods Phys. Res. Sect. B Beam Interact. Mater. Atoms.

[11] Apel P., Blonskaya I., Oganessian V., Orelovitch O., Trautmann C. (2001). Morphology of latent and etched heavy ion tracks in radiation resistant polymers polyimide and poly(ethylene naphthalate). Nucl. Instrum. Methods Phys. Res. Sect. B Beam Interact. Mater. Atoms.

[12] Apel P.Y., Blonskaya I.V., Orelovitch O.L., Sartowska B.A., Spohr R. (2012). Asymmetric ion track nanopores for sensor technology. Reconstruction of pore profile from conductometric measurements. Nanotechnology.

[13] Apel P.Y., Ramirez P., Blonskaya I.V., Orelovitch O.L., Sartowska B.A. (2014). Accurate characterization of single track-etched, conical nanopores. Phys. Chem. Chem. Phys..

[14] Blonskaya I.V., Kristavchuk O.V., Nechaev A.N., Orelovich O.L., Polezhaeva O.A., Apel P.Y. (2020). Observation of latent ion tracks in semicrystalline polymers by scanning electron microscopy. J. Appl. Polym. Sci..

[15] Abu Saleh S., Eyal Y. (2004). Porous tracks along wakes of swift uranium ions in polyimide. Appl. Phys. Lett..

[16] Abu Saleh S., Eyal Y. (2005). Morphology of track cores and halos created by swift uranium ions in polycarbonate. Nucl. Instruments Methods Phys. Res. Sect. B Beam Interact. Mater. Atoms.

[17] Sessler G.M. (1987). Electrets.

[18] Camacho Gonzalez F., Mellinger A., Gerhard-Multhaupt R. Energy levels of charge traps in polyethylene terephthalate films. Proceedings of the 2004 International Conference on Solid Dielectrics.

[19] Ouchi I. (1983). Anisotropic Absorption and Reflection Spectra of Poly(ethylene terephthalate) Films in Ultraviolet Region. Polym. J..

[20] LaFemina J.P., Arjavalingam G. (1991). Photophysics of poly(ethylene terephthalate): Ultraviolet absorption and emission. J. Phys. Chem..

[21] Takai Y., Osawa T., Mizutani T., Ieda M. (1977). Photoconduction in poly(ethylene terephthalate). I. Mechanisms of carrier generation. J. Polym. Sci. Polym. Phys. Ed..

[22] Takai Y., Mori K., Mizutani T., Ieda M. (1978). Field quenching of thermoluminescence from photoexcited polyethylene tereph-thalate (PET). J. Phys. D Appl. Phys..

[23] Kressmann R., Sessler G., Gunther P. (1996). Space-charge electrets. IEEE Trans. Dielectr. Electr. Insul..

[24] Fleischer R.L., Price P.B., Walker R.M. (1975). Nuclear Tracks in Solids.

[25] Kilic A., Russell S., Shim E., Pourdeyhimi B. (2017). The charging and stability of electret filters. Fibrous Filter Media.

[26] Zdorovets M., Kozlovskiy A., Harrison F., Tuleushev A. (2020). Induced ordering in polyethylene terephthalate films irradiated with Ar ions with an energy of 70 MeV. Surf. Coat. Technol..

[27] Temnov D., Rossouw A., Vinogradov I., Shabanova N., Mamonova T., Lizunov N., Perold W., Nechaev A. (2021). Thermo-activation spectroscopy of track-etched membranes based on polyethylene terephthalate films irradiated by swift Xe ions. Radiat. Phys. Chem..

[28] Daubeny R.d.P., Bunn C.W. (1954). The crystal structure of polyethylene terephthalate. Proc. R. Soc. Lond. Ser. A Math. Phys. Sci..

[29] Amborsky L.E. (1962). Structural dependence of the electrical conductivity of polyethylene terephthalate. J. Polym. Sci..

[30] Tuleushev A.Z., Zdorovets M.V., Kozlovskiy A.L., Harrison F.E. (2020). Ion charge influence on the molecular structure of polyethylene terephthalate films after irradiation with swift heavy ions. Crystals.

[31] Ström P., Primetzhofe D. (2021). Energy deposition by nonequilibrium charge states of MeV 127I in Au. Phys. Rev..

[32] Tuleushev A.Z., Zdorovets M.V., Kozlovskiy A.L., Harrison F.E. (2020). Induced Spirals in Polyethylene Terephthalate Films Irradiated with Ar Ions with an Energy of 70 MeV. Crystals.

[33] Liu C., Zhu Z., Jin Y., Sun Y., Hou M., Wang Z., Wang Y., Zhang C., Chen X., Liu J. (2000). Study of effects in polyeth-ylene terephthalate films induced by high energy Ar ion irradiation. Nucl. Instrum. Methods Phys. Res. B.

[34] Apel P.Y., Blonskaya I.V., Ivanov O.M., Kristavchuk O.V., Lizunov N.E., Nechaev A.N., Orelovich O.L., Polezhaeva O.A., Dmitriev S.N. (2020). Creation of Ion-Selective Membranes from Polyethylene Terephthalate Films Irradiated with Heavy Ions: Critical Parameters of the Process. Membr. Membr. Technol..

[35] Tuleushev A.Z., Harrison F.E., Kozlovskiy A.L., Zdorovets M.V. (2021). Assessment of the Irradiation Exposure of PET Film with Swift Heavy Ions Using the Interference-Free Transmission UV-Vis Transmission Spectra. Polymers.

[36] Roberts G.G., Keating B.S., Shelley A.V. (1974). Electroabsorption in disordered solids: Selenium. J. Phys. C Solid State Phys..

[37] Roberts G.G., Keating B.S., Vincett P.S., Barlow W.A. (1978). Electroabsorption in disordered solids. II. Anthracene crystals and thin films. J. Phys. C Solid State Phys..

[38] Ziegler J.F., Biersack J.P., Ziegler M.D. (2009). The Stopping and Range of Ions in Matter.

[39] Seanor D.A. (1982). Electrical Properties of Polymers.

[40] Gorokhovatsky Y., Temnov D., Marat-Mendesa J.N., Dias C.J.M., Das-Gupta D.K. (1998). On the nature of thermally stimulat-ed discharge current spectra in polyethylene terephthalate. J. Appl. Phys..

[41] Swanepoel R. (1983). Determination of the thickness and optical constants of amorphous silicon. J. Phys. E Sci. Instrum..

[42] Swanepoel R. (1984). Determination of surface roughness and optical constants of inhomogeneous amorphous silicon films. J. Phys. E Sci. Instrum..

[43] Evans R.M. (1948). An Introduction to Color.

[44] Tuleushev A.Z., Harrison F.E., Kozlovskiy A.L., Zdorovets M.V. (2022). Urbach Rule in the Red-Shifted Absorption Edge of PET Films Irradiated with Swift Heavy Ions. Polymers.

[45] Kizel V.A. (1973). Reflection of Light.

[46] Cardona M. (1969). Modulation Spectroscopy.

[47] Klyava Y.G. (1985). The Urbach rule and continual disorder in noncrystalline solids. Sov. Phys. Solid State.

[48] Tauc J., Menth A. (1972). States in the gap. J. Non-Cryst. Solids.

[49] Wood D.L., Tauc J. (1972). Weak Absorption Tails in Amorphous Semiconductors. Phys. Rev. B.

[50] Nurmukhametov R.N. (1971). Absorption and Luminescence of Aromatic Compounds.

[51] Tribelsky M.I., Miroshnichenko A.E. (2022). Resonant scattering of electromagnetic waves by small metal particles: A new insight into the old problem. Physics-Uspekhi.

[52] Dow J.D., Redfield D. (1970). Electroabsorption in Semiconductors: The Excitonic Absorption Edge. Phys. Rev. B.

[53] Teyssèdre G., Menegotto J., Laurent C. (2001). Temperature dependence of the photoluminescence in poly(ethylene terephthalate) films. Polymer.

[54] Takai Y., Mori K., Mizutani T., Ieda M. (1977). Study on Electron Traps in Polyethylene Terephthalate by Thermally Stimulated Current and Photo-Stimulated Detrapping Current Analyses. Jpn. J. Appl. Phys..

[55] Bonch-Bruevich V.L. (1983). Problems of the electron theory of disordered semiconductors. Sov. Phys. Uspekhi.

[56] Mott N.F., Davis E.A. (2012). Electronic Processes in Noncrystalline Materials.

[57] Dow J.D., Redfield D. (1971). Theory of Exponential Absorption Edges in Ionic and Covalent Solids. Phys. Rev. Lett..

[58] Dow J.D., Redfield D. (1972). Toward a Unified Theory of Urbach’s Rule and Exponential Absorption Edges. Phys. Rev. B.

[59] Franz W. (1958). Einfluß eines elektrischen Feldes auf eine optische Absorptionskante. Z. Naturforsch..

[60] Olley J. (1973). Structural disorder and the urbach edge. Solid State Commun..

[61] Vrehen Q.H.F. (1966). Interband Optical Absorption in Crossed Electric and Magnetic Fields in Germanium. Phys. Rev..

[62] Wentzel E.S. (1982). Probability Theory.

[63] Diaz A.F., Felix-Navarro R.M. (2004). A semi-quantitative tribo-electric series for polymeric materials: The influence of chemical structure and properties. J. Electrost..

[64] Reichardt C., Welton T. (2002). Solvents and Solvent Effects in Organic Chemistry.

[65] McNaught A.D., Wilkinson A., IUPAC (1997). Compendium of Chemical Terminology (The “Gold Book”).

[66] Gridchin V., Pucklyakov Y., Shurman L. (1992). Variband GaAs_(1 − x)_ P_x_: A material for pressure sensors. Sens. Actuators A Phys..

[67] Saidov A.S., Leiderman A.Y., Karshiev A.B. (2019). Photothermovoltaic Effect in a Si_x_Ge_1–x_ Variband Solid Solution. Appl. Sol. Energy.

[68] Apel P.Y., Fink D. (2004). Ion-track etching. Transport Processes in Ion-Irradiated Polymers.

[69] Blonskaya I., Kirilkin N., Kristavchuk O., Lizunov N., Mityukhin S., Orelovich O., Polezhaeva O., Apel P. (2023). Visualization and characterization of ion latent tracks in semicrystalline polymers by FESEM. Nucl. Instrum. Methods Phys. Res. Sect. B Beam Interact. Mater. Atoms.

[70] Chernov A.A., Trusov L.I. (1969). Electrostatic effects in the formation of nuclei (seeds) at the surface. Sov. Phys. Crystallogr..

[71] Trusov L.I., Kholmyansky V.A. (1973). Island Metal Films.

[72] Distler G.I., Vlasov V.P., Gerasimov J.M., Kobzareva S.A., Kortukova E.I., Lebedeva E.N., Moskvin V.V., Shenjavskaja L.A. (1976). Decorating the Surface of Solids.

[73] Hashimoto Y., Takeuchi S., Sunaoshi T., Yamazawa Y. (2018). Voltage Contrast Imaging with Energy-Controlled Signal in an FE-SEM. Microsc. Microanal..

